# Examining the Relationship Between Gastroschisis and Placental Fetal Vascular Malperfusion

**DOI:** 10.1177/10935266211029629

**Published:** 2021-07-21

**Authors:** Brittany Ruschkowski, Ahmed Nasr, Irina Oltean, Sarah Lawrence, Dina El Demellawy

**Affiliations:** 1 Faculty of Medicine, University of Ottawa, Ottawa, Ontario, Canada; 2 Department of Pediatric Surgery, Children’s Hospital of Eastern Ontario, Ottawa, Ontario, Canada; 3 Department of Pediatrics, Neonatology Division, Children’s Hospital of Eastern Ontario, Ottawa, Ontario, Canada; 4 Department of Pathology, Children’s Hospital of Eastern Ontario, Ottawa, Ontario, Canada

**Keywords:** basic research, clinical neonatology, dysmorphology, fetal, GI, microscopy, neonatal, placenta, teratology, vascular

## Abstract

**Introduction:**

Gastroschisis is a congenital malformation characterized by intestinal herniation through an abdominal wall defect. Despite its unknown pathogenesis, known risk factors include maternal smoking, alcohol use, and young maternal age. Previous work has shown that gastroschisis is associated with placental delayed villous maturation, and the goal of this study was to assess for additional associated placental pathologies that may help clarify the pathogenesis of gastroschisis.

**Methods:**

We conducted a retrospective slide review of 29 placentas of neonates with gastroschisis. Additionally, we reviewed pathology reports from one control group of 30 placentas with other congenital malformations. Gross and histological data were collected based on a standardized rubric.

**Results:**

Gastroschisis was associated with increased placental fetal vascular malperfusion (FVM) in 62% of cases (versus 0% of controls, p < 0.0001). It was also associated with increased placental villous maldevelopment in 76% of cases (versus 3% of controls, p < 0.0001).

**Conclusion:**

Our study demonstrates an association between gastroschisis and FVM. While FVM could be the consequence of vascular disruption due to the ventral location of gastroschisis, it could also reflect estrogen-induced thrombosis in early pregnancy. Further research is needed to separate these possibilities and determine the cause of the placental FVM observed in gastroschisis.

## Background

Gastroschisis is a congenital malformation characterized by herniation of abdominal organs into the amniotic cavity through a defect in the fetal anterior abdominal wall. The prevalence of gastroschisis is 4.19 per 10,000 total births in Canada,^1^ and multiple studies suggest that rates of gastroschisis are increasing both in Canada and worldwide.^2–7^ Gastroschisis is associated with significant neonatal morbidity and mortality, with almost all affected neonates experiencing comorbidities including feeding intolerance and failure to thrive.^8^ Mortality rates are reported at almost 10 percent, even in cases with appropriate obstetric and neonatal care.^9,10^

Although gastroschisis is often discussed in the context of other fetal abdominal wall defects such as omphalocele, gastroschisis is unique in that the prolapse nearly always occurs to the right of an otherwise normal umbilicus, and the prolapsed abdominal contents have no membranous covering.^11^ Because of this lack of a membranous covering, unlike in omphalocele, the intestines are directly exposed to amniotic fluid, leading to increased bowel damage thought to be caused by inflammatory proteins and digestive compounds in the amniotic fluid.^12^ As compared to omphalocele, gastroschisis is less likely to be associated with other malformations and chromosomal abnormalities.^13^

While the etiology and pathogenesis of gastroschisis remains unclear, there are several known risk factors including maternal smoking and alcohol use, low socioeconomic status, and inadequate prenatal care.^14^ Current literature identifies additional risk factors, including specific gene variants^15^ and genitourinary infection in early pregnancy.^16^ Further, young maternal age has long been shown to be a significant risk factor – for example, a 2007 European study^5^ found that compared to mothers aged 25–29 years, the risk of gastroschisis was increased in mothers aged under 25 years, and especially in those under 20 years.

Numerous theories have been proposed to explain the pathogenesis of gastroschisis, including failed incorporation of the yolk sac into the umbilical stalk,^17^ abnormal folding of the ventral body wall,^18^ and abnormal involution of the right umbilical vein.^19^ A more recent hypothesis by Lubinsky^20^ postulates that gastroschisis is the result of an estrogen-induced thrombotic event in early pregnancy. Specifically, Lubinsky’s theory posits that palmitic acid byproducts of thrombosis interfere with early cell signaling, prior to the fusion of the body wall folds, leading to the abdominal wall defect seen in gastroschisis. This theory could help to clarify young maternal age as a risk factor for gastroschisis, as first trimester estrogen levels are higher in mothers of younger age, thus predisposing them to estrogen-induced thrombosis. Further, adverse health behaviours such as smoking and alcohol use (known gastroschisis risk factors) may also elevate estrogen levels in premenopausal women, thereby increasing their risk of gastroschisis.^21^,^22^ However, despite this and other more recent hypotheses, the pathogenesis of gastroschisis remains an overall controversial topic in the literature, and the majority of existing theories are limited in that they do not account for the increasing prevalence of gastroschisis, its predominantly right-sided location, or its strong association with younger maternal age.

Additionally, multiple studies have shown that gastroschisis is associated with increased risk of spontaneous preterm birth,^23^,^24^ with a recent study reporting 8.5 percent of its 1421 cases having spontaneous delivery before 34 weeks of gestational age (GA).^25^ The reason for this is not entirely clear but is likely multifactorial. One such factor may be that the main risk factors for gastroschisis, including maternal smoking and young maternal age, are also known risk factors for preterm birth.^26^,^27^ However, even after adjusting for factors that differ between neonates with and without gastroschisis, the risk of preterm birth remains elevated, suggesting that this is not the sole explanation.^25^ We believe that examining the placental pathology associated with gastroschisis, especially in cases with preterm birth, could contribute to a better understanding of the underlying factors contributing to this increased risk.

Regarding placental correlates of gastroschisis, the literature is scarce; however, a 1985 study by Ariel and Landing described the presence of uniform amniocyte vacuolization in 3 of 4 reviewed gastroschisis cases.^28^ Additionally, our group recently conducted a study that showed an association between gastroschisis and placental delayed villous maturation (DVM),^29^ and a 2014 abstract by Cox and Popek described findings of placental fetal thrombotic vasculopathy in 5 reviewed gastroschisis cases.^30^ Of note, in 2015, the term *fetal vascular malperfusion* (FVM) was introduced by the Amsterdam International Consensus Group of placental pathologists to encompass lesions previously described by terms including *fetal vascular obstructive lesions*, *fetal vascular thrombi*, and *fetal thrombotic vasculopathy*.^31–33^ FVM refers to a group of placental lesions that are the consequence of reduced or absent fetal perfusion of the villous parenchyma, most commonly caused by umbilical cord obstruction leading to stasis, ischemia, and sometimes thrombosis.^34^

A better understanding of the placental correlates of gastroschisis would help to elucidate its pathogenesis. Therefore, the purpose of this study was twofold: first, to identify any additional placental pathologies associated with gastroschisis, which may provide indications to its pathogenesis; and second, to determine how maternal age and fetal GA at birth may influence placental pathology in the context of gastroschisis.

## Materials and Methods

With approval from our institutional review board, we conducted a retrospective slide review of placentas of cases with gastroschisis at the Children’s Hospital of Eastern Ontario (CHEO) between October 2013 and December 2019. All selected cases had a diagnosis of gastroschisis provided in the clinical history section of the pathology report. Excluded cases included those with missing slides and those cases with pathology reports indicating that the placenta was received by the lab in formalin, rather than fresh. At our institution, the majority of placentas are received fresh, and we wanted to ensure standard fixation and processing conditions across cases and controls.

A limited neonatal chart review was also conducted for all gastroschisis cases. Data collected included whether the gastroschisis was an isolated defect versus associated with other congenital anomalies, and in cases associated with other anomalies, these were specified. Relevant maternal and fetal data was also collected from the clinical history section of the pathology reports corresponding to gastroschisis cases. Information collected included maternal smoking, maternal diabetes, fetal intrauterine growth restriction (IUGR), and intrauterine fetal death (IUFD).

We selected one control group of placentas with congenital malformations other than gastroschisis including cardiac, pulmonary, gastrointestinal, genitourinary, brain, and extremity malformations (Table 1). This control group did not include cases with congenital malformations in the context of other potential confounders affecting placental pathology, such as maternal diabetes, hypertension, smoking, or substance use.^35–37^ This control group was maternal age-matched and fetal GA-matched to the case group. Placentas in the control group were selected across the same time period (October 2013 to December 2019) as the gastroschisis cases. All placentas from gastroschisis cases were reviewed by a perinatal pathologist (DD). Slides were assessed for all pathologies using a standardized placenta data collection rubric developed and validated by our team.^38^ Definitions of lesions outlined in this rubric are based on corresponding definitions from the Amsterdam consensus statement.^31^ As per institutional protocols, slides from five tissue blocks were examined: one from the umbilical cord, one from the membrane roll, and three from the placental disc. For any additional localized lesions, tissue was added to the umbilical cord block. If this was not possible due to lack of space, additional blocks were added. For control group placentas, histological data were collected from the corresponding original pathology reports and graded using this same rubric.

**Table 1.** Breakdown by Organ System of Congenital Malformations Seen in Control Group.

Organ System	N	Percentage of Cases (%)	Specific Malformations (n)
Cardiac	7	23.3	Hypoplastic left heart syndrome (2), pulmonary valve hypoplasia (2), pulmonary atresia (1), ASD (1), VSD (1), Tetralogy of Fallot (1)
CNS	6	20.0	Absent corpus callosum (3), Dandy-Walker malformation (2), anencephaly (1)
Respiratory	6	20.0	Congenital diaphragmatic hernia (3), pulmonary hypoplasia (1), congenital pulmonary airway malformation (1), tracheoesophageal fistula (1)
GU	5	16.7	Hypospadias (1), duplex kidney (1), polycystic kidney disease (1), bilateral renal agenesis (1), left renal agenesis (1)
GI	4	13.3	Omphalocele (2), anal atresia (1), esophageal atresia (1)
ENT	3	10.0	Cleft lip and palate (1), isolated cleft lip (1), ear malformation (1)
MSK	2	6.7	Bilateral club feet (1), polydactyly (1)

Note that several control placentas were from cases with multiple malformations. ASD, atrial septal defect; VSD, ventricular septal defect; CNS, central nervous system; GU, genitourinary; GI, gastrointestinal; ENT, ear/nose/throat; MSK, musculoskeletal.

For data processing purposes, each placenta was assigned a binary score to represent the presence (1) or absence of FVM (0). A score of 1 was assigned if the placenta met criteria for the presence of any of the following histological features: avascular fibrotic villi, fetal vascular thrombosis, intramural fibrin deposition, villous stromal-vascular karyorrhexis, or stem villous vascular obliteration. Each placenta with features of FVM was then classified as having a segmental (FVM features seen on one slide only) versus global FVM pattern (multifocal findings of FVM, seen on more than one slide). Further, the FVM for each case was classified as either low- or high-grade as per the Amsterdam consensus statement criteria.^31^ Similar to FVM grading, each placenta was also assigned a binary score for placenta villous maldevelopment (VMD). A score of 1 for this criterion was assigned if the placenta demonstrated any of the following histological features: chorangiosis, chorangiomas, or DVM.

Statistical analysis comparing the assessment of FVM and VMD between the gastroschisis and control group, the frequency of FVM and VMD among cases of younger (<20 years) versus older maternal age (≥20 years), frequency of FVM and VMD in preterm (<37 weeks GA) versus term (≥37 weeks GA) gastroschisis cases, and frequency of FVM and VMD in cases of isolated versus non-isolated gastroschisis, were performed using Fisher’s exact test (GraphPad Software, La Jolla, CA).

## Results

Twenty-nine placentas from gastroschisis cases, with mean GA of 35 weeks (SD 3.9) and mean maternal age of 24 years (SD 4.3), were examined. Average GA and maternal age of the control group placentas (n = 30) were 37 weeks and 27 years, respectively. In the gastroschisis group, 5 placentas were from cases with maternal age less than 20 years old, while 24 placentas originated from cases with maternal age at least 20 years old. There were 20 preterm cases versus 9 term cases.

**Figure 1.**
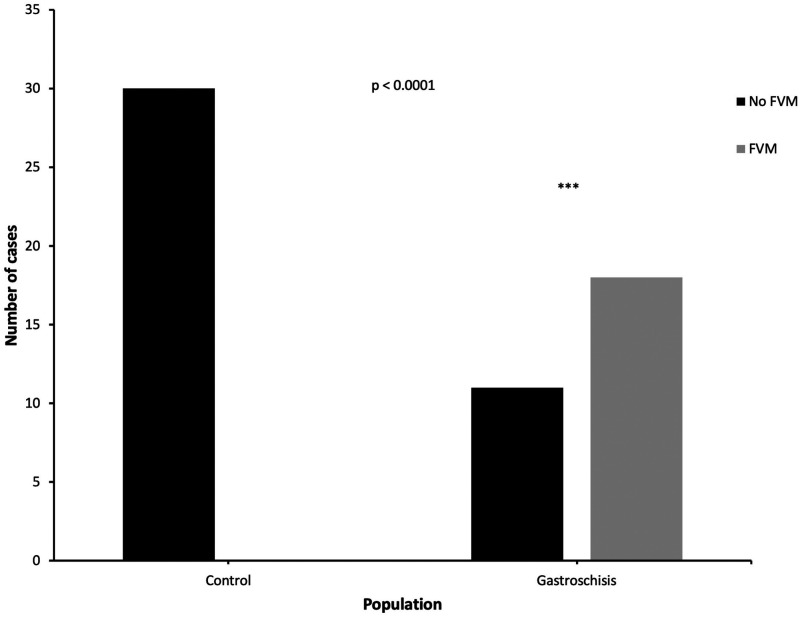
FVM in control versus gastroschisis groups. Fisher’s exact test was used to compare findings of FVM between control versus gastroschisis groups. FVM, fetal vascular malperfusion.

**Figure 2.**
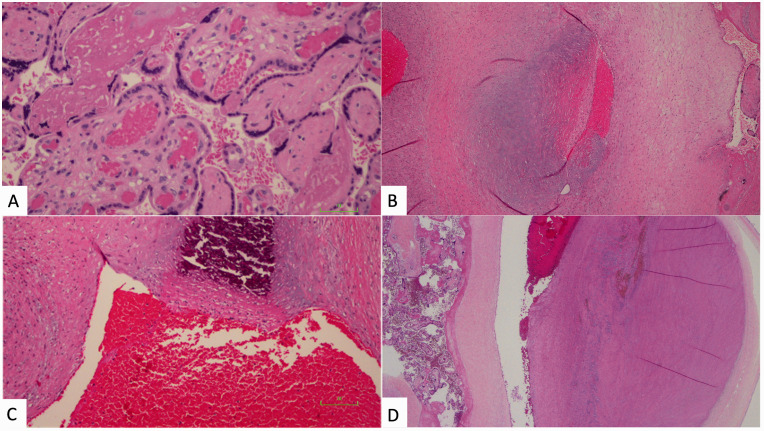
Histologic images of placentas from gastroschisis cases, showing features of FVM. A, Avascular fibrotic villi (right) seen beside normal vascularized villi (center) (hematoxylin and eosin, 400X magnification). B, Chorionic plate vessel with acute non-occlusive intramural fibrin deposition within a focus of connective tissue (hematoxylin and eosin, 100X magnification). C, Chorionic plate vessel with intramural fibrin deposition and calcification (hematoxylin and eosin, 400X magnification). D, Dilated chorionic plate vessel with acute non-occluding thrombus (hematoxylin and eosin, 40X magnification).

In the gastroschisis group, 18/29 cases (62%) demonstrated at least one feature of FVM versus 0/30 cases (0%) from the control group (*P* < 0.0001, Figure 1). Cases exhibited foci of avascular fibrotic villi (n = 7), fetal vascular thrombosis (n = 10), intramural fibrin deposition within fetal vessels (n = 10), villous stromal-vascular karyorrhexis (n = 2), and stem villous vascular obliteration (n = 2). Figure 2 demonstrates representative histology. Five of these 18 cases demonstrated global FVM. The remainder of the cases with FVM (n = 13) demonstrated segmental FVM. Four gastroschisis cases met the Amsterdam criteria for high-grade FVM; the remainder of cases (n = 25) were classified as low-grade. Table 2 outlines a breakdown of the specific FVM findings seen in each gastroschisis case. No gastroschisis cases had gross umbilical cord abnormalities such as true knots or hypercoiling.

**Table 2.** Breakdown by Case of Histologic FVM Findings Seen in Gastroschisis Cases.

Case	FVM Findings
1	Small foci of avascular villi, one old occlusive thrombus in chorionic plate vessel
2	Small foci of avascular villi
3	Focal acute non-occlusive thrombus in chorionic plate vessel
**4**	**Two acute non-occlusive thrombi in chorionic plate vessels, recent intramural fibrin deposition in two chorionic plate vessels, villous stromal-vascular karyorrhexis**
5	Recent intramural fibrin deposition in a stem vessel
**6**	**Large foci of avascular villi, one acute occlusive thrombus in a stem vessel, recent intramural fibrin deposition in a stem vessel**
7	Recent intramural fibrin deposition in one chorionic plate vessel
8	One acute non-occlusive thrombus in a stem vessel
9	One acute non-occlusive thrombus in a chorionic plate vessel, stem villous vascular obliteration
**10**	**Large foci of avascular villi, two acute non-occlusive thrombi in chorionic plate vessels, intramural fibrin deposition in four fetal vessels (three recent, one remote)**
11	Intermediate foci of avascular villi, one acute non-occlusive thrombus in a stem vessel, intramural fibrin deposition in one chorionic plate vessel, stem villous vascular obliteration
12	Small foci of avascular villi, one acute non-occlusive thrombus in a stem vessel, intramural fibrin deposition in one fetal vessel
13	Villous stromal-vascular karyorrhexis
14	Recent intramural fibrin deposition in one fetal vessel
**15**	**Large foci of avascular villi**
16	Recent intramural fibrin deposition in one stem vessel
17	One acute non-occlusive thrombus in chorionic plate vessel
18	Recent intramural fibrin deposition in two stem vessels

Cases meeting criteria for high-grade FVM are bolded. FVM, fetal vascular malperfusion. Note that large foci of avascular villi refers to over 45 avascular villi in total.

**Figure 3.**
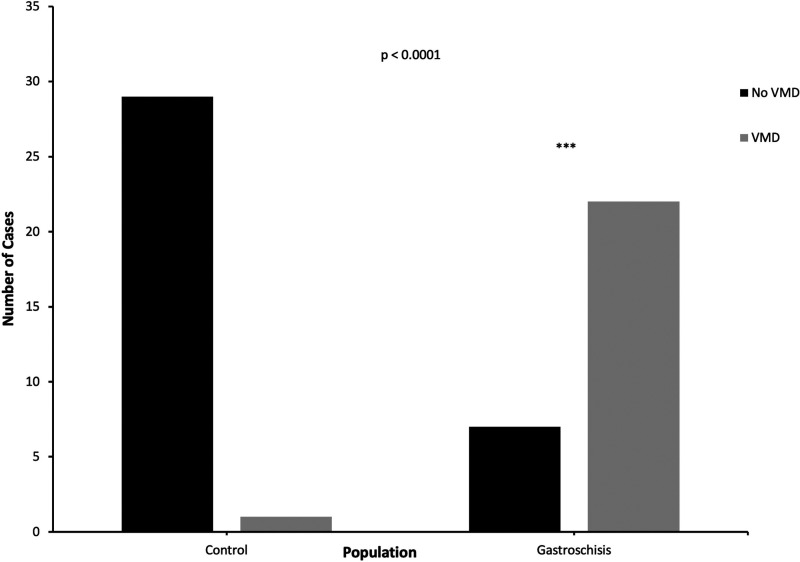
VMD in control versus gastroschisis groups. Fisher’s exact test was used to compare findings of placental VMD between control versus gastroschisis groups. VMD, villous maldevelopment.

**Figure 4.**
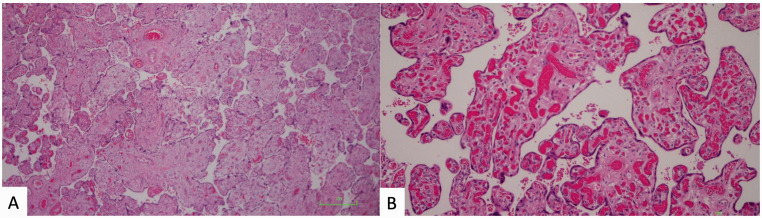
Histologic images of placentas from gastroschisis cases, showing features of placental VMD. A, Villi from a gastroschisis case at 38 weeks gestational age, showing DVM (hematoxylin and eosin, 100X magnification). B, Villi from a gastroschisis case, showing chorangiosis (hematoxylin and eosin, 200X magnification).

Features of placental VMD were present in 22/29 gastroschisis cases (75.9%) versus 1/30 cases (3.3%) from the control group (*P* < 0.0001, Figure 3). Twenty gastroschisis cases showed DVM and 10 cases had chorangiosis (Figure 4). No gastroschisis cases had chorangiomas. The control case with VMD, a placenta from a neonate with pulmonary atresia, showed mild patchy chorangiosis only. In the gastroschisis group, 13/29 cases (44.8%) demonstrated features of both FVM and VMD, versus 0/30 cases (0%) from the control group.

**Figure 5.**
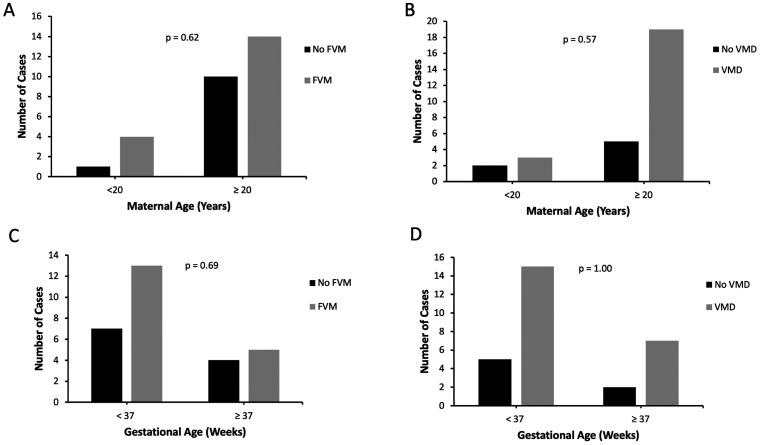
A, FVM in gastroschisis cases with maternal age <20 years versus ≥20 years. B, VMD in gastroschisis cases with maternal age <20 years versus ≥20 years. Fisher’s exact test was used to compare findings of FVM and VMD between cases with maternal age <20 years versus ≥20 years. C, FVM in preterm (GA < 37 weeks) versus term (GA ≥37 weeks) gastroschisis cases. D, VMD in preterm (GA < 37 weeks) versus term (GA ≥37 weeks) gastroschisis cases. Fisher’s exact test was used to compare findings of FVM and VMD between preterm and term gastroschisis cases. FVM, fetal vascular malperfusion. VMD, villous maldevelopment.

In the gastroschisis group, there was no statistically significant difference in frequency of FVM in cases with maternal age < 20 years compared to cases with maternal age ≥20 years (80.0% versus 58.3%, p = 0.62, Figure 5(A)). There was also no significant difference in frequency of VMD in gastroschisis cases with young versus older maternal age (60.0% versus 79.2%, p = 0.57, Figure 5(B)). Similarly, there was no significant difference in frequency of FVM or VMD in preterm versus term gastroschisis cases (65.0% versus 55.5%, p = 0.69; and 75.0% versus 77.8%, p = 1.00, respectively, Figure 5(C) and (D)).

Aside from features of FVM and VMD, amniocyte vacuolization was seen in 16/30 (53.3%) gastroschisis cases. Other gross and histologic findings in gastroschisis cases included villous fibrinoid necrosis (n = 1), retroplacental hematoma (n = 4), increased focal perivillous fibrin deposition (n = 1), intervillous thrombi (n = 3), chronic deciduitis (n = 7), and amnion nodosum (n = 3). Features of maternal vascular malperfusion were occasionally seen, with one gastroschisis case each showing focal villous agglutination, focal distal villous hypoplasia, subacute placental infarct, and increased syncytial knots. Two gastroschisis cases showed evidence of ascending intrauterine infection, and of these cases, 1/2 (50.0%) had features of FVM. Three gastroschisis cases showed chronic villitis. Of these three cases, 2/3 (66.7%) showed no features of FVM, and the remaining 1/3 (33.3%) showed high-grade FVM, with findings including avascular villi, thrombosis, and intramural fibrin deposition. Notably, this case showed only one focus of low-grade chronic villitis that was not associated with the avascular villi seen.

Regarding clinical data, in the gastroschisis group, maternal smoking was present in 11/29 cases (37.9%) and maternal diabetes was present in 1/29 cases (3.4%). No cases had both maternal smoking and maternal diabetes. Of the cases with maternal smoking, 7/11 (63.6%) showed features of FVM (3 high-grade and 4 low-grade), and 10/11 (90.9%) showed features of VMD. The case with maternal diabetes had features of both FVM (high-grade) and VMD.

In the gastroschisis group, 2/29 cases (6.9%) resulted in IUFD and 1/29 cases (3.4%) resulted in spontaneous abortion at 17 weeks GA. Of these three cases, all three had findings of VMD but only 1/3 cases (33.3%) had findings of FVM. The remaining 26 cases were live births. 5/29 cases (17.2%) were associated with IUGR. Of these 5 cases, 3/5 (60.0%) showed features of both FVM and VMD, with one case having features of FVM only.

**Figure 6.**
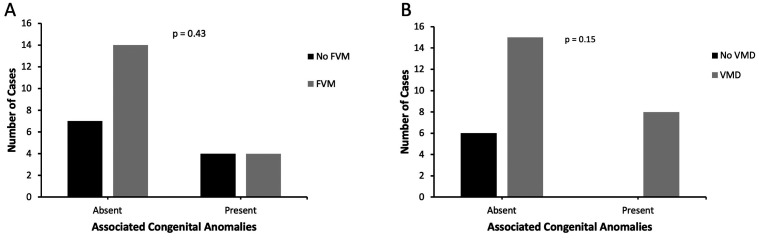
A, FVM in cases of isolated versus non-isolated gastroschisis. B, VMD in cases of isolated versus non-isolated gastroschisis. Fisher’s exact test was used to compare findings of FVM and VMD in cases of isolated versus non-isolated gastroschisis.

Eight gastroschisis cases (27.6%) were associated with other major congenital anomalies (Table 3). In the remaining 21 cases, additional congenital anomalies including hemangioma (n = 1), grade I hydronephrosis (n = 1), torticollis (n = 1), cryptorchidism (n = 3), congenital hypothyroidism (n = 1), and short long bones (isolated and interpreted as constitutional, n = 2) were identified but interpreted as minor congenital anomalies, and therefore for purposes of data analysis they were considered as cases of isolated gastroschisis. There was no statistically significant difference in frequency of FVM or VMD in cases with isolated versus non-isolated gastroschisis (66.7% versus 50.0%, p = 0.43; and 71.4% versus 100.0%, p = 0.15, respectively, Figure 6). Of the four cases associated with small intestinal atresia, all had findings of placental VMD but only 2/4 cases (50.0%) showed placental FVM, one high-grade and one low-grade. Of the remaining three gastroschisis cases with high-grade FVM, one other case had associated major congenital anomalies (hypospadias, congenital hydrocele, and mesenchymal hamartoma), one case had associated minor anomalies only (bilateral cryptorchidism and congenital hypothyroidism), and the final case was of entirely isolated gastroschisis.

**Table 3.** Breakdown by Case of Associated Congenital Malformations Seen in Gastroschisis Cases.

Case	Specific Congenital Malformations
4	Hypospadias, hepatic mesenchymal hamartoma, hydrocele
15	Small intestinal atresia (type IIIB), bilateral cryptorchidism
16	Small intestinal atresia (jejunal)
18	Dysplastic, multi-cystic right kidney with duplex collecting system
19	Small intestinal atresia with microcolon
20	Small intestinal atresia with microcolon, congenital torticollis, grade I hydronephrosis
21	Syndactyly, polysyndactyly, congenital torticollis
22	Ventricular septal defect, short long bones

Case numbers correspond to those in Table 2. Note that cases 19–22 did not have histologic findings of FVM and therefore do not have corresponding findings in Table 2.

## Discussion

Regarding placental correlates of gastroschisis, literature is limited to the 1985 Ariel and Landing study showing amniocyte vacuolization,^28^ an abstract by Cox and Popek describing findings of FVM,^30^ and a study by our group showing placental DVM.^29^ In this study, we assessed placentas from cases of gastroschisis using a standardized placenta data collection rubric^38^ with the goal of identifying any additional associated placental findings. We similarly used this rubric to collect histological data from a group of control placentas (cases with other congenital malformations).

Overall, we observed a statistically significant increase in frequency of placental VMD among gastroschisis cases versus controls, thereby supporting the previously established link between gastroschisis and these placental findings.^29^ As outlined in our previous work,^29^ we believe that the association between gastroschisis and VMD may be due to: (1) a common upstream factor contributing to the development of both gastroschisis and DVM, likely nutritional and/or geographical given the association between gastroschisis and maternal undernutrition,^39^,^40^ and epidemiological data showing geographical clustering of gastroschisis cases;^41^,^42^ or (2) villous maldevelopment due to alterations in the placental and amniotic environment secondary to gastroschisis. Because maternal diabetes, the predominant maternal condition associated with placental DVM,^37^ was only present in one gastroschisis case, we are confident that this is a true association and not a finding related to potential maternal confounders.

Moreover, similar to the findings of Cox and Popek,^30^ we observed increased rates of placental FVM in gastroschisis cases. This is the first study that has described this association between gastroschisis and placental FVM in these numbers – the existing literature examining these outcomes has much smaller sample sizes (i.e., 5 cases)^30^ due to the low incidence of gastroschisis in general. One potential explanation for this association is that FVM, as proposed by Cox and Popek,^30^ is a consequence of the ventral location of gastroschisis, causing umbilical cord disruption with subsequent thrombosis. Supporting this theory is the fact that the predominant risk factor for FVM is obstruction of blood flow in the umbilical cord vessels,^34^ particularly the umbilical vein.^43^ This obstruction can be caused by pathologies including umbilical cord deformities (i.e. strictures, knots, or hypercoiling) or pressure on the umbilical cord from prolapse or entanglement.^34^ Thus, the ventral location of gastroschisis could similarly cause compromise or disruption of the umbilical cord vessels, resulting in thrombosis and other downstream findings of FVM. Of note, additional gross umbilical cord abnormalities (i.e., potential confounders) in our study are unlikely, since none of our cases exhibited true umbilical cord knots or hypercoiling.

In contrast, if Lubinsky’s hypothesis^20^ is correct, this FVM could be a reflection of early pregnancy estrogen-induced thrombosis that leads to gastroschisis, rather than a secondary consequence of vascular disruption by herniated abdominal organs as suggested by Cox and Popek.^30^ Of note, in terms of placental correlates, Lubinsky’s hypothesis also highlights the palmitic acid byproducts of thrombosis, which could not only interfere with cell signaling in early development,^17^,^18^ causing the abdominal wall defect seen in gastroschisis, but could also be the source of the unique amniocyte vacuoles commonly seen in gastroschisis cases since their first description by Ariel and Landing.^28^ Supporting Lubinsky’s theory is the fact that the contents of these vacuoles have been examined and indeed were found to be rich in palmitic acid.^44^ Ultimately, further investigation is needed to determine the etiology of the FVM seen in gastroschisis cases, and it is possible that elements of both hypotheses are playing a role.

In our analysis of the neonatal charts, we found that the majority (21/29) of our gastroschisis cases were not associated with other major congenital anomalies. This is consistent with the well-established notion that gastroschisis typically occurs as an isolated malformation.^13^ When comparing cases of isolated versus non-isolated gastroschisis, we did not observe a statistically significant difference in rates of FVM or VMD. This suggests that even in the minority of gastroschisis cases that are associated with other major congenital anomalies, it does not appear that these anomalies are driving the placental pathology seen in gastroschisis. Interestingly, in the four gastroschisis cases with associated small intestinal atresia, only two showed findings of placental FVM. Therefore, despite the fact that small intestinal atresia is thought to be due to in utero vascular compromise that causes ischemic injury,^45^ this ischemic process does not appear to consistently result in placental FVM. Future larger-scale studies examining placental pathology in intestinal atresia are required to better understand the effect of this vascular accident on the placenta.

## Limitations

The main limitation of our analysis is that while all slides from gastroschisis cases were reviewed by a perinatal pathologist during our data collection period, the histological data for our control group placentas was collected from the original pathology reports rather than from contemporaneous slide review. Therefore, our study team was not blinded to which placentas were from cases of gastroschisis versus controls. Despite this limitation, we feel that our association still stands, particularly because each gastroschisis case was carefully analyzed using an exhaustive, previously validated rubric. This rubric was used to comprehensively assess each gastroschisis placenta for all possible pathologies rather than just FVM findings. Additionally, although it is possible that some minor histological findings were missed or not reported by the original pathologist assigned to each control case, many of our gastroschisis cases with FVM showed either multiple FVM features or multivessel involvement, which if present in a control case would be extremely unlikely to be omitted from the original pathology report. Another potential limitation is our inclusion criteria for the control group, defined as healthy mothers with uncomplicated pregnancies with congenital malformations, other than gastroschisis. We did not have access to placentas from completely normal healthy controls; therefore, our controls are only an approximation of a true control, and lack generalizability. However, rarely are true control placentas sent to the pathologist for review at our institution.

A second limitation of our analysis is that detailed correlation with maternal diseases that could predispose to placental FVM, such as maternal thrombophilias,^46^ was not performed. However, regarding maternal thrombophilias, even the most common thrombophilia, Factor V Leiden, has quite low prevalence (as low as 0.45 percent depending on ethnic origin);^47^ and to our knowledge, there is no literature establishing maternal thrombophilias as a risk factor for gastroschisis. Because 60 percent of the cases in our study showed features of FVM, this association almost certainly exists independently of maternal thrombophilias.

In addition, regarding statistical analysis, we are restricted by our relatively small sample size of 30 gastroschisis cases. By comparing placental pathology in gastroschisis cases from younger (<20 years) versus older (≥20 years) mothers, we had hoped to identify differences in pathology between these age groups, thereby providing novel insights into why younger pregnant women are disproportionately affected by gastroschisis. We similarly hoped to identify differences in placental pathology from term versus preterm gastroschisis cases to provide insight into why gastroschisis cases have an increased risk of spontaneous preterm birth. However, statistical power in comparing placental pathology in gastroschisis cases of younger versus older maternal age, as well as term versus preterm gastroschisis cases, was limited; hence, we do not know whether an association is truly lacking or rather if it could not be detected. Importantly, as aforementioned, the existing literature in this area generally has much smaller sample sizes due to the low incidence of gastroschisis. Future, large scale and multi-disciplinary studies examining the effect of maternal age and fetal GA on placental pathology in gastroschisis are encouraged.

## Future Directions

In addition to larger-scale studies, as a future direction, a detailed neonatal chart review would be helpful to determine if the placental pathology seen in gastroschisis (FVM and VMD) is associated with adverse clinical outcomes. Another relevant future direction for this study would be to conduct a large-scale slide review of placentas from younger mothers without neonates with gastroschisis and compare these to placentas from mothers of older age. This investigation would help to determine if features of VMD and FVM are more common in younger mothers in general compared to mothers of older age, in the absence of any congenital anomalies such as gastroschisis. Finally, a more clinical future direction could be to measure serum estrogen levels in pregnant women at the time of gastroschisis diagnosis and compare this with serum estrogen levels in controls, in order to determine if gastroschisis cases do in fact show higher early pregnancy estrogen levels, which would support Lubinsky’s hypothesis.

## Conclusion

Our study shows an association between gastroschisis and FVM. The FVM seen in our study could reflect thrombosis as a result of vascular compromise due to the ventral location of gastroschisis, or it could be a reflection of estrogen-dependent maternal thrombosis. Ultimately, further investigation is needed to clarify these and other hypotheses surrounding the pathogenesis of gastroschisis. This study lays the necessary groundwork for future investigation and serves as a reminder of the potential role of placental pathology in understanding the pathogenesis of gastroschisis.

Although we did not identify differences in placental pathology between gastroschisis cases from younger versus older mothers, nor in term versus preterm gastroschisis cases, our statistical power was limited by our relatively small sample size. Therefore, we are unable to determine whether an association is truly lacking or if it instead simply could not be detected. We recommend further large scale multi-disciplinary studies to clarify the effect of maternal age and fetal GA on placental pathology in gastroschisis.
